# Health Care Analytics Challenges: A 3-Pillar Framework Connecting Analytics Maturity, Workforce Agility, and Technical Enablement

**DOI:** 10.2196/96541

**Published:** 2026-08-31

**Authors:** Samuel Thomas Harrold

**Affiliations:** 1 Yuimedi, Inc., 240 Elm Street, 2nd Floor, Somerville, MA, 02144, United States, 1 512-516-3464

**Keywords:** institutional amnesia, organizational forgetting, human in the loop, medical informatics, sociotechnical systems, query governance, natural language processing, knowledge management, personnel turnover, organizational resilience, artificial intelligence, AI

## Abstract

Health care organizations face a “triple threat” of low analytics maturity, high workforce instability, and semantic technical barriers that together produce a crisis of “institutional amnesia.” Leadership turnover, workforce shortages, and widespread intent to leave among informatics specialists systematically erase the tacit knowledge required to navigate complex clinical data schemas, trapping organizations in a cycle where knowledge loss outpaces knowledge capture. Viewed through the socialization, externalization, combination, and internalization model of knowledge creation by Nonaka, the root cause is a “socialization failure”: high turnover fractures the social networks required for mentorship, rendering the traditional apprenticeship model of informatics unsustainable. To address this failure, we used a design science research approach synthesizing evidence from health care informatics, knowledge management, and natural language processing to develop a sociotechnical framework: human-in-the-loop knowledge governance (HITL-KG). HITL-KG is designed to shift the locus of organizational knowledge from volatile human memory to durable semantic artifacts called “validated query triples,” each comprising a natural language intent, executable SQL, and rationale metadata. By embedding knowledge capture into the daily query workflow, the framework aims to convert ephemeral analytics into permanent institutional assets. The accompanying 3-pillar assessment rubric enables organizations to identify compounding vulnerabilities across analytics maturity, workforce agility, and technical enablement. The “validator paradox” (who validates the AI when experts leave?) is addressed by reframing validation through lean “standard work”: each validated query establishes the current known standard rather than eternal truth, functioning as a “knowledge ratchet” that prevents regression. Decoupling analytical capability from individual tenure lets analytics maturity advance even as the workforce evolves. This paper proposes and theoretically motivates the framework; empirical validation is deferred to a companion study.

## The Triple Threat: Institutional Amnesia in Health Care Analytics

The health care analytics landscape is currently paralyzed by a “triple threat” of compounding failures: (1) persistently *low analytics maturity*, where despite decades of investment, as of late 2024, only 39 organizations worldwide had reached the top tiers of the Healthcare Information and Management Systems Society (HIMSS) Analytics Maturity Assessment Model (AMAM) [1,2]; (2) a *semantic gap* between clinical intent and technical schema implementation [3,4]; and (3) a profound crisis of *workforce instability* that creates “institutional amnesia” [5].

While technical barriers and maturity models are well documented, the workforce dimension has shifted from a management concern to an existential threat. Modern longitudinal data on analytics staff are fragmented, but the available signals are alarming. As of 2024, a total of 53% of health care chief information officers have held their roles for less than 3 years [6], creating a strategic vacuum at the top. At the operational level, global nurse turnover runs at 16% to 18% annually [7,8], and a 2025 study found that 55% of public health informatics specialists intend to leave their positions [9].

This turnover creates what public administration scholars term “institutional amnesia” [10,11] and organization science studies refer to as “organizational forgetting” [12,13]: the systematic erasure of the tacit knowledge required to interpret complex health data in health care analytics. In health care, “data” are never raw; they are wrapped in layers of institutional context (billing rules, workflow work-arounds, and unwritten exclusions) [14]. When the analyst who knows that “exclusion code 99” actually means “hospice transfer” leaves, that knowledge evaporates. The organization does not just lose an employee; it loses the ability to accurately measure its own performance.

Current literature approaches these problems in isolation. Analytics maturity models (eg, the HIMSS AMAM) assume a stable workforce capable of linear progression [1]. Technical solutions (eg, natural language to SQL; NL2SQL) assume a stable schema and clear intent [15]. Neither accounts for the reality of the “great resignation,” where the rate of knowledge loss often exceeds the rate of knowledge capture [13].

Traditional knowledge management strategies (wikis, data dictionaries, and documentation) have failed because they are *passive* [16]. They require overworked staff to stop working and write down what they know. In a high-burnout environment, this documentation is the first casualty. As a result, health care systems are trapped in a Sisyphus-like cycle: hiring new analysts who spend their limited tenure relearning the same institutional secrets only to leave just as they become productive [17,18]. A foundational 2004 study established that technical IT staff in organizations where IT is a support function, as in health care delivery, had the lowest expected tenure of any organizational category at just 2.9 years [19]. That this 2-decade–old study remains a key benchmark is itself evidence of the crisis: the industry lacks the stability to track its own attrition. Contemporary signals, detailed below, suggest that the situation has worsened.

This viewpoint addresses a critical sociotechnical gap: *how can health systems maintain analytics maturity when workforce turnover exceeds the speed of documentation?*

As a viewpoint, this paper deliberately advances a prescriptive position: that health care organizations should shift from passive knowledge management to active, artifact-based governance. The analysis below is grounded in descriptive evidence of why current approaches fail, but the architectural recommendations are intentionally directive.

We propose that the solution lies not in better documentation but in a fundamental architectural shift: moving from *passive* knowledge management to *human-in-the-loop knowledge governance* (HITL-KG).

## Theoretical Grounding: Socialization, Externalization, Combination, and Internalization and the Unstable Workforce

### Overview

We ground our analysis in the socialization, externalization, combination, and internalization model of knowledge creation by Nonaka [20], informed by a narrative literature review across health care analytics maturity, workforce turnover, and natural language processing, with gray literature assessed using the authority, accuracy, coverage, objectivity, date, and significance checklist [21]. Gray literature was retained only when no peer-reviewed equivalent existed or when it provided unique industry data. The model describes organizational knowledge as emerging through a continuous cycle of 4 conversion modes:

1. *Socialization* (tacit to tacit)—knowledge transfers through shared experience and co-located practice, as when a senior analyst teaches a junior colleague the unwritten rules of a clinical dataset.
2. *Externalization* (tacit to explicit)—individuals articulate tacit know-how into explicit forms, such as documents or coded artifacts, as when an analyst records why a specific exclusion code maps to hospice transfers.
3. *Combination* (explicit to explicit)—separately documented knowledge is integrated and systematized into broader structures, as when data dictionary entries are consolidated into a governed analytics catalog.
4. *Internalization* (explicit to tacit)—individuals learn from documented knowledge and convert it into personal expertise through practice, as when a new analyst studies validated query libraries.

In a healthy organization, these 4 modes form a self-reinforcing spiral: tacit insights become documented, documentation becomes systematized, and systematized knowledge is internalized by new members who then generate fresh tacit insights [20]. When any mode breaks down, the spiral stalls. In health care analytics, the breakdown is at the very first stage.

The framework’s 3 pillars name the organizational capabilities at stake in the “triple threat”: analytics maturity, workforce agility, and technical enablement. This structure aligns with established models across health care informatics and knowledge management (Table 1).

**Table 1.** Framework alignment with established models.

Pillar	HIMSS^a^ AMAM^b^ alignment	DIKW^c^ hierarchy	Knowledge management
Analytics maturity	Progression from stages 0-7	Data→information	Organizational learning
Workforce agility	Implicit in advanced stages	Knowledge (tacit)→wisdom	Tacit knowledge transfer
Technical enablement	Stages 6-7 requirements	Information→knowledge	Knowledge codification

a HIMSS: Healthcare Information and Management Systems Society.

b AMAM: Analytics Maturity Assessment Model.

c DIKW: data, information, knowledge, and wisdom.

The HIMSS AMAM provides organizational benchmarks but does not address workforce knowledge retention. The data, information, knowledge, and wisdom hierarchy explains progression from raw data to actionable insights but does not account for institutional memory loss. The 3-pillar framework synthesizes these perspectives, positioning workforce dynamics as the critical enabler connecting data access (analytics maturity) with organizational wisdom (knowledge preservation) [13,20].

### The Broken Cycle: Socialization Failure

In the model by Nonaka, *socialization* is the foundational conversion mode: the primary channel through which newcomers absorb tacit context that formal training cannot convey [20]. Socialization depends on 2 preconditions: sustained interaction and sufficient temporal overlap between knowledge holders and receivers [22]. It is, in effect, an apprenticeship model requiring years of shared practice.

In the current health care environment, this mechanism has collapsed. The leadership and operational turnover documented above reveals an “apprenticeship window” shorter than the knowledge transfer cycle it must support: 30% of new employees depart within their first year [23], whereas specialized informatics roles require 18 to 24 months to reach fluency [17]. The arithmetic is unforgiving: by the time a new analyst has absorbed enough tacit context to be productive, their mentor may already be gone, and the new analyst is themselves halfway through an average tenure.

High turnover rates fracture the social networks required for mentorship [7,8]. The resulting knowledge loss is compounding: each departure removes a node from the organization’s informal knowledge network, making subsequent socialization attempts less effective because fewer experienced practitioners remain to serve as mentors [24]. Socialization is no longer a viable strategy for resilience.

### The Solution: Externalization via Sociotechnical Artifacts

To survive, organizations must shift reliance from socialization to *externalization*: converting tacit knowledge into explicit, durable artifacts [25]. However, traditional externalization (writing wikis, maintaining data dictionaries, and composing runbooks) suffers from 2 critical weaknesses. First, it is passive: it requires overworked staff to interrupt their workflow and perform a separate documentation task [26]. In a high-burnout environment where organizations face persistent attrition and talent shortages [27], this discretionary documentation is the first casualty. Second, it is low fidelity: the act of writing down tacit knowledge inevitably loses nuance, context, and the conditional logic that makes institutional knowledge valuable [22]. The result is documentation that exists but does not adequately capture what the departing expert actually knew.

We propose a form of *active* externalization: one that captures tacit knowledge as a by-product of the daily analytics workflow rather than a separate documentation burden. The mechanism is a new sociotechnical artifact: the *validated query triple* (see Multimedia Appendix 1 for worked examples). This artifact consists of (1) *natural language intent* (the clinical business question, eg, “Hypertension readmissions excluding planned transfers”), (2) *executable SQL* (the technical implementation), and (3) *rationale metadata* (the “why” behind the logic, eg, “Excluding status 02 per Centers for Medicare and Medicaid Services (CMS) 2025 rule”).

By capturing these 3 components *during the act of analytics*, we transform the ephemeral work of query generation into a permanent institutional asset [28].

## HITL-KG

We propose HITL-KG as the overarching governance framework, extending the knowledge governance approach [29] with the validated query cycle as its core operational process. This framing reflects that the system serves as a governance mechanism, not just a productivity tool.

### The HITL-KG Architecture

**Figure 1.**
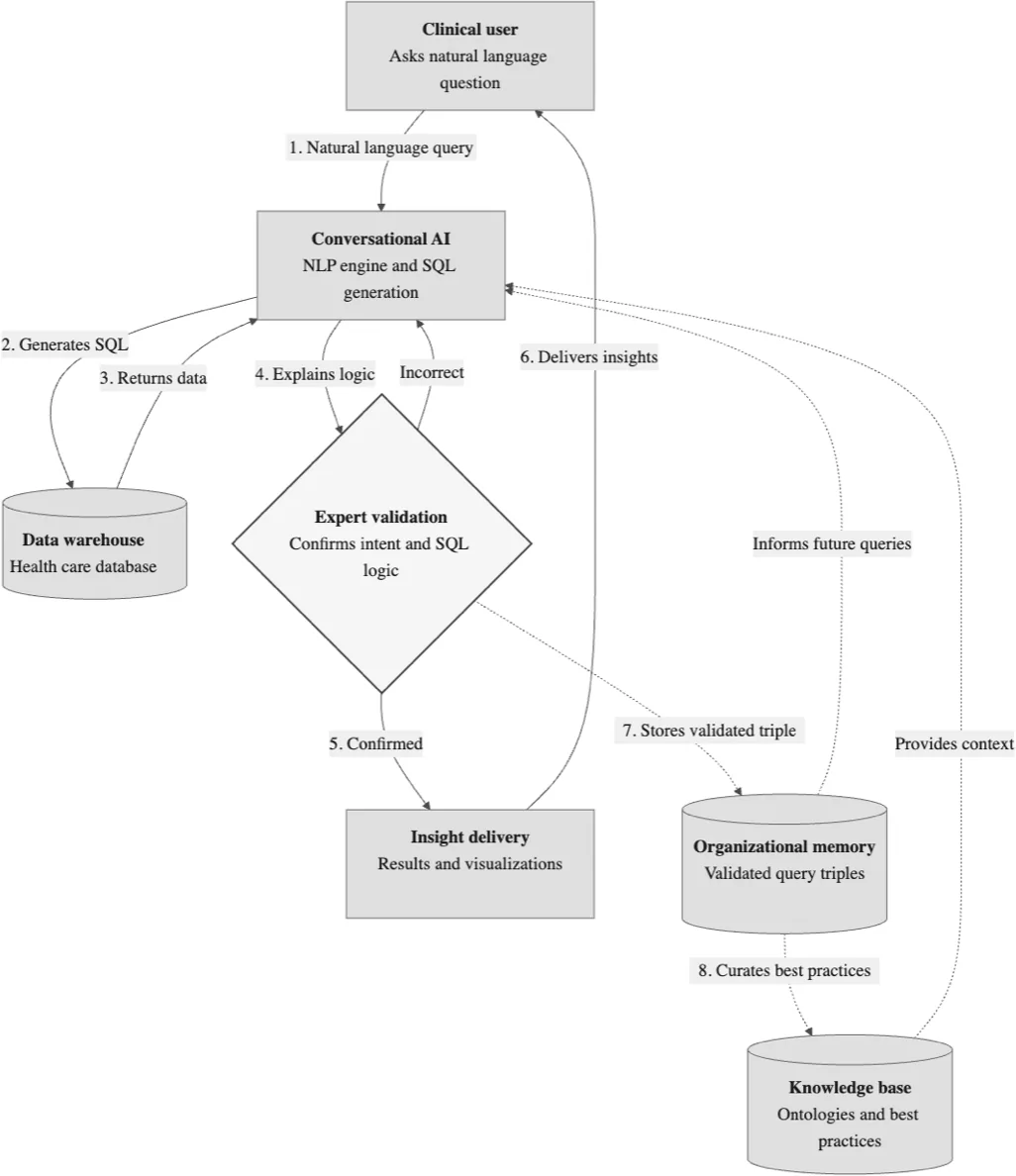
Human-in-the-loop knowledge governance architecture. NLP: natural language processing.

The HITL-KG architecture (Figure 1) functions as a *governance forcing function*. It inserts a mandatory validation step into the analytics workflow, preventing laundered hallucinations while capturing expert knowledge.

A clinical user’s natural language query (step 1) is translated to SQL and run against the data warehouse (steps 2-3); an expert validation gate (step 4) either returns confirmed insights (steps 5-6) or loops back for correction. Confirmed query triples enter organizational memory (step 7; dashed line in Figure 1), which informs future queries and curates the knowledge base (step 8), closing a continuous learning loop in which best practices evolve rather than remain static.

**Figure 2.**
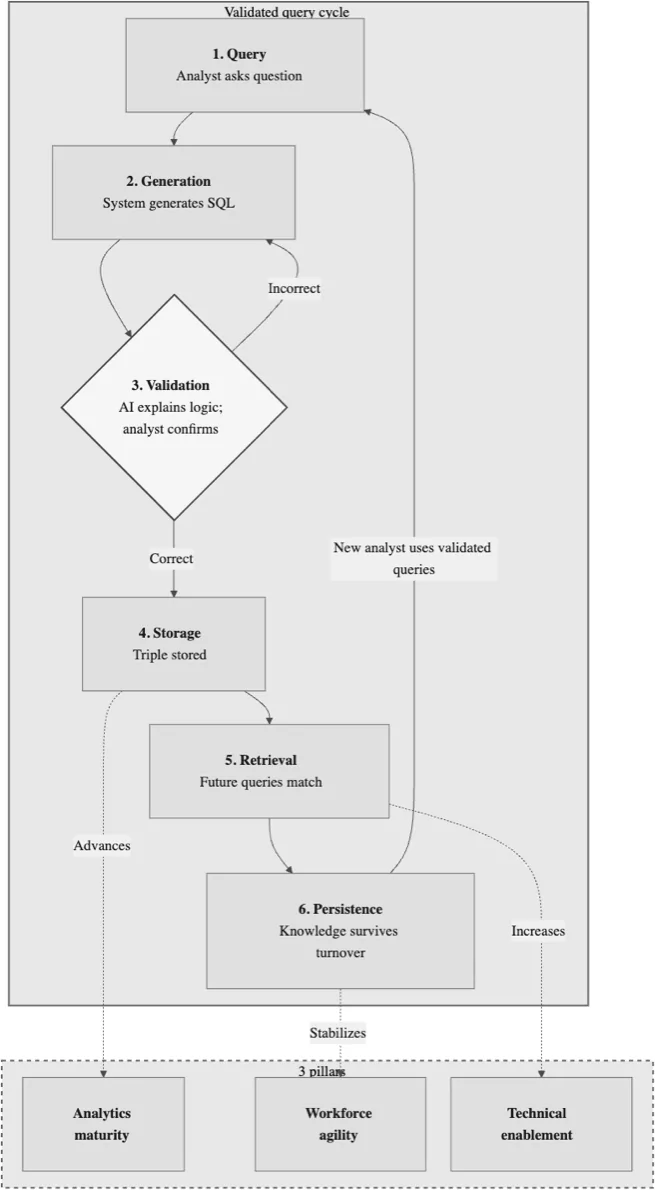
The 6-step validated query cycle and its mapping to the 3 pillars.

Figure 2 summarizes the corresponding 6-step validated query cycle, tracing queries from clinical intent to expert validation into durable organizational memory.

A natural language question (step 1) yields a candidate SQL (step 2) that the analyst validates against the system’s explained logic (step 3), looping back if incorrect. Validated triples are stored in organizational memory (step 4) matched by future queries (step 5) and persist across staff turnover (step 6). The dashed arrows in Figure 2 mark the pillar outcomes: storage advances analytics maturity, persistence stabilizes workforce agility, and retrieval increases technical enablement.

### The Process of Externalization

The cycle proceeds from query generation, in which a conversational AI system translates the user’s natural language question into candidate SQL [30], to *expert validation*, the critical moment of externalization where the domain expert confirms or corrects the AI’s interpretation and thereby captures tacit knowledge [31,32]. The validated triple is hashed and stored in organizational memory [33], then retrieved ahead of future probabilistic generation [34]. Multimedia Appendix 1 details each step alongside worked examples.

### Comparison With Existing Approaches

Organizations have addressed institutional memory loss through several strategies, each with limitations that HITL-KG overcomes.

*Code-based semantic layers* (eg, data build tool and LookML) encode business logic in version-controlled repositories but suffer from “schema rot,” where electronic medical record (EMR) data models change frequently and maintenance exceeds high-turnover teams’ capacity, misaligning the layer and the underlying data [35]. HITL-KG’s validated query triples share the version control principle but add the rationale metadata that semantic layers lack: not just *what* a query does but *why*.

*Traditional knowledge management* (wikis, data dictionaries, and runbooks) relies on passive capture, where users must stop working to document, which reduces participation and produces inaccurate records under cognitive load [16,26]. Such documentation might suffice for a small, stable query set, but real workloads are neither: more than half of analytical queries recur in large production workloads [36], yet even small databases log hundreds to thousands of distinct query strings [37], so a fixed set of documented queries captures neither the large recurring core nor the evolving ad hoc tail. HITL-KG instead implements active capture across both regimes: the query itself is the documentation, validated at the point of use [28].

*Unsupervised AI querying* removes human oversight entirely; current NL2SQL accuracy makes this unsafe for clinical analytics [38]. HITL-KG occupies the middle ground: AI generates, humans validate.

## The Evidence Base: 3 Pillars

### Overview

The evidence presented below supports the existence and severity of the problem that HITL-KG addresses and the maturity of the technologies it requires. It does not constitute empirical validation of the framework itself, which remains a theoretically grounded, testable proposition whose empirical evaluation is the subject of future work.

The HITL-KG framework is supported by 3 pillars of empirical evidence synthesized from the literature cited throughout this paper.

### Pillar 1: Analytics Maturity Evidence

*Analytics maturity* describes an organization’s progression in using data and quantitative models for fact-based decisions [39], a construct operationalized by staged maturity models that extend beyond the HIMSS AMAM [40,41]. Health care maturity remains chronically low. Assessments reveal that only 26 organizations worldwide achieved AMAM stage 6 and 13 reached stage 7 by late 2024 [1,2]. Because AMAM assessment is voluntary and self-selected, these figures likely overrepresent analytics-committed organizations, so low maturity may be even more widespread than the counts suggest [42,43]. The corollary is that the vast majority of organizations remain at the lower stages, characterized by fragmented data and limited predictive capabilities [44]. However, maturity is not merely an IT metric; it is a clinical safety predictor. In HIMSS’s companion, the EMR adoption model, which measures the EMR adoption underlying analytics capability, levels 6 and 7 correlate with 3.25 times higher odds of better Leapfrog Hospital Safety Grades [45]. Low maturity creates a “low maturity trap” where data quality issues (such as the 39%-71% missing data rates in cancer databases [46]) remain uncorrected because the experts who understand the context are leaving.

Critically, low maturity is not simply a status; it is a self-reinforcing trap. Organizations at stages 0 to 3 lack the automated monitoring and data governance infrastructure needed to detect their own deficiencies, so poor data quality goes unrecognized. The clinical consequences are measurable: one Medicare accountable care organization that implemented analytics to overcome electronic health record fragmentation reduced readmission rates from 24% to 17.8% and achieved US $1.6 million in cost savings [47]. However, data interoperability remains a leading obstacle to analytics adoption [3,4]. Barriers including employee resistance to change and lack of organizational readiness further stall data-driven initiatives [48,49]. The trap thus compounds: each year of delayed investment widens the gap between what the organization could know and what it does know.

### Pillar 2: Workforce Agility Evidence

*Workforce agility* is a workforce’s capacity for proactivity, adaptability, and resilience under change [50,51]. In health care analytics, the evidence below documents the instability that systematically erodes this capacity. The cost of turnover in informatics is higher than in standard IT. Knowledge loss can cost up to 3 times the annual salary [24,52]. In clinical informatics specifically, replacement costs range from US $50,000 to US $230,000 per departing employee, with a loss of 39 staff members in a single department in 1 year incurring a total cost of up to US $8.97 million [53]. With 30% of new employees leaving within their first year [23], health care IT professionals spend a limited portion of their employment at full productivity as specialized roles require 18 to 24 months to reach fluency [17]. This “revolving door” prevents the accumulation of the “collective knowledge structures” required for complex task performance [13].

The workforce crisis operates at multiple reinforcing levels, as detailed in the Theoretical Grounding: Socialization, Externalization, Combination, and Internalization and the Unstable Workforce section: leadership churn, operational shortages, and foundational attrition collectively prevent the sustained mentorship that socialization requires. This multilevel instability ensures that the temporal overlap between experienced practitioners and newcomers rarely materializes.

### Pillar 3: Technical Enablement Evidence

*Technical enablement* denotes the technologies and practices that let nonspecialist domain users access and analyze data without heavy reliance on central IT [54,55]. NL2SQL technology has matured along a clear accuracy gradient: general-purpose models achieve approximately 65% execution accuracy on health care–specific benchmarks such as MIMICSQL [56], domain-adapted systems such as MedT5SQL reach 80% accuracy [57], and architecturally specialized approaches combining large language models (LLMs) with structured knowledge representations achieve 94% accuracy on the same benchmarks [58]. While insufficient for unsupervised clinical deployment [38], this gradient demonstrates that the generation engine required for HITL-KG is viable within a human-validated workflow. Notably, high benchmark accuracy does not guarantee real-world generalization: evaluation on more challenging dataset splits reveals accuracy drops from 92% to 28% [59], reinforcing the necessity of the human-in-the-loop architecture. Health care–specific natural language interfaces such as Criteria2Query achieve fully automated cohort query formulation in 1.22 seconds per criterion [60], with over 80% of clinical users indicating willingness to adopt such tools.

However, NL2SQL is an enabler, not a solution in itself. Its deeper significance lies in the organizational prerequisites it demands. For an AI system to translate a natural language question into a correct SQL query, the organization must first establish validated data models, explicit business logic definitions, and codified domain terminology [3,4]. This reframes NL2SQL from a convenience tool into a catalyst for the kind of systematic knowledge externalization that the HITL-KG framework requires. The interface bridges the semantic gap between clinical experts and technical schema, allowing nontechnical domain experts to interact with data alongside broader modernization efforts [61-64]. The very difficulty of making NL2SQL work correctly thus becomes a governance opportunity: it compels organizations to surface and formalize tacit knowledge that would otherwise depart with its experts.

## Organizational Self-Assessment

### Overview

To operationalize the framework, we propose a *3-pillar assessment rubric* (Table 2) that enables health care organizations to evaluate their current position across each pillar and identify compounding vulnerabilities.

**Table 2.** Three-pillar assessment rubric indicators (the full rubric with anchors can be found in Multimedia Appendix 2).

Pillar	Indicators
Analytics maturity	HIMSS^a^ AMAM^b^ stage, self-service analytics, and AI and NL^c^ interface
Workforce agility	First-year analytics staff turnover, leadership tenure, and knowledge concentration
Technical enablement	Data access, interoperability, and schema coupling

a HIMSS: Healthcare Information and Management Systems Society.

b AMAM: Analytics Maturity Assessment Model.

c NL: natural language.

### Why Assessment, Not Just Maturity

As noted, the maturity models and knowledge management frameworks in Table 1 assume linear progression by a stable workforce [1]. In practice, an organization that reaches AMAM stage 5 but regresses to stage 3 after the departure of 2 senior analysts has not failed to mature; it has failed to be *resilient*. A recent systematic review of organizational resilience measurement in health care found no consensus on what to measure [65]. Only 4 instruments have been developed specifically for health care, and only 2 have been validated [66]. The 3-pillar assessment rubric addresses this gap by organizing evidence-based indicators around the domains where institutional amnesia operates, measuring not just where an organization stands but also how vulnerable it is.

### The 3-Pillar Rubric

Each indicator is scored as being of low, medium, or high strength using evidence-based anchors from the literature reviewed in the The Evidence Base: 3 Pillars section. Organizations scoring predominantly as “low strength” across multiple pillars face the self-reinforcing degradation cycle that the framework identifies as the central threat. Table 2 summarizes the 9 indicators; the full rubric with scoring anchors and evidence can be found in Multimedia Appendix 2.

The “schema coupling” indicator can be operationalized through *continuous analytic integration*: treating validated query triples as software assets within a continuous integration and continuous delivery pipeline that detects data and schema drift [35,67,68]. When a data warehouse schema is updated, the system automatically reruns stored queries and flags failures, transforming institutional memory into a living test suite [69,70].

The rubric complements rather than replaces the AMAM. Where the AMAM measures the sophistication of analytical capabilities at a point in time, the 3-pillar assessment rubric reveals how vulnerable those capabilities are to the compounding effects of turnover, low maturity, and technical barriers. Used together, they provide a 2D view of analytics health.

## The Validator Paradox and Standard Work

A critical objection to HITL-KG is circular: if the framework requires domain experts to validate AI-generated queries and the core problem is that domain experts are leaving, then the framework fails precisely when it is most needed. This *validator paradox* represents the strongest counterargument to the approach proposed here, and addressing it requires moving beyond simplistic reassurance.

The resolution draws on lean management’s concept of “standard work” [71]. In this framing, validation is not the establishment of *eternal truth* but the documentation of the *current known standard*. Each validated query triple records the best available understanding of how a business question maps to a data operation at that moment; the validation is time-stamped and contextual, not permanent. Critically, as Alukal and Manos [71] establish, standard work is the prerequisite for kaizen (continuous improvement): without a documented baseline, there is no foundation to improve upon. Each validated query, therefore, establishes a floor, not a ceiling. The next expert, veteran or midcareer hire, inherits a baseline to refine rather than reconstructing institutional knowledge from scratch.

This mechanism functions as what we term a *knowledge ratchet*, consistent with findings on collective knowledge structures [13]. Each validated triple prevents regression below the last confirmed state. Even if a subsequent validator is less experienced, the organization cannot slide below the previously validated standard. The ratchet does not guarantee forward progress, but it prevents the catastrophic backsliding that is the central failure mode of institutional amnesia.

Real-world evidence supports this: UC Davis Health moved from AMAM stage 0 to stage 6 by establishing standardized “S.M.A.R.T.” definitions for its analytics metrics [72]. Those codified standards survived staff turnover because they existed as organizational artifacts rather than knowledge held solely by their creators. The HITL-KG validated query library serves an analogous function: it encodes analytical decisions into durable, retrievable structures that persist independent of any single analyst’s tenure.

The validator paradox is not fully resolved: below a minimum viable expertise threshold, validation becomes meaningless. A junior analyst rubber-stamping AI-generated output without genuine comprehension provides no knowledge ratchet; the validation artifact exists, but its epistemic value is nil. Identifying this threshold remains an open empirical question; future work should measure it via controlled hallucination injection studies in which AI-generated queries containing deliberate errors are presented to validators of varying experience levels.

We propose a provisional 3-tier governance model informed by risk-stratified AI oversight frameworks [73,74] to degrade gracefully when expertise is scarce. The first tier is *full validation*: a domain expert with schema-level knowledge reviews the query triple and confirms semantic alignment (the default). The second tier is *constrained validation*: when no fully qualified validator is available, a less experienced analyst reviews the triple against previously validated queries for the same data domain, flagging deviations for deferred expert review; the triple is stored with “provisional” status. The third tier is *automated matching*: for queries that match an existing validated triple with high semantic similarity, the system accepts the match without human review, logging it for periodic audit.

Organizational governance requirements include who can validate queries (domain expertise thresholds), review workflows for high-stakes queries, query versioning as schemas evolve, and retrieval policies. Drawing on established master data governance practice [75], organizations can designate specific validated triples as “golden queries” certified by a governance committee as the authoritative source of truth for key metrics. Only these certified triples serve as official standards, mitigating shadow IT risks [76] while preserving analytical agility.

## Safety as Cognitive Forcing

HITL-KG is fundamentally a *safety mechanism*, not a productivity tool. The central risk of unsupervised AI in clinical analytics is what we term “laundering hallucinations”: a plausible-sounding but factually incorrect query result that enters the decision pipeline undetected and influences clinical or operational choices. Because LLMs generate fluent, confident output regardless of correctness, errors arrive dressed in the language of expertise, harder to catch than obviously malformed output.

HITL-KG mitigates this risk through *cognitive forcing functions* [38], a design pattern borrowed from clinical decision-making and safety engineering. Requiring the AI to explain its reasoning *before* presenting results forces the validator into system 2 (analytical and deliberate) thinking rather than system 1 (fast and heuristic) acceptance of superficially plausible outputs. User studies confirm the practical benefit: structured explanation reduces error recovery time by 30 to 40 seconds compared to unstructured output review [77].

Critically, the human validator’s value is not merely assumed. Recent studies show that human oversight measurably improves AI-generated analytics: LLM output verified by a reviewer outperformed a human-only workflow (91.0% vs 89.0% accuracy) across 6 systematic reviews [78], and a text-to-SQL validation gate in which experts corrected generated queries improved semantic correctness by 28.4% over end-to-end model output [79]. These gains are conditional on well-designed interaction [80]; the comparative effectiveness of HITL-KG itself remains the subject of the planned empirical validation.

The parallel to aviation safety is instructive: checklists and mandatory callouts made commercial aviation exceptionally safe not by eliminating human error but by surfacing errors before they propagate. HITL-KG applies the same principle: the mandatory validation step is an analytical “callout” that interrupts uncritical acceptance so that the friction of validation is not a cost but the mechanism of safety itself.

## Structural Barriers: Why the Problem Persists

Failed standardization approaches (eg, IBM Watson Health [81,82] and Haven [83,84]) demonstrate that centralized models fail clinical reality. Metadata uncertainties and “messy” institution-specific business logic require localized solutions [3,82]. HITL-KG addresses this by capturing *local* logic rather than enforcing *global* standards.

## Limitations

This work is a narrative, design science–informed framework rather than a systematic review or multisite empirical evaluation. The literature base is concentrated on English-language sources and recent (2024-2026) workforce and NL2SQL studies, so findings may not capture all regional, specialty-specific, or technological contexts. The HITL-KG architecture and the proposed 3-pillar assessment rubric are conceptual artifacts that require future implementation and validation in diverse health systems before their effectiveness and generalizability can be fully established.

HITL-KG also complements rather than replaces workforce development: it decouples institutional knowledge from individual tenure but still presumes investment in the training and analytics literacy through which staff learn to pose, validate, and interpret queries [17]. This complementarity also bounds the framework. Where data models and business rules change continually, no approach is self-maintaining; precisely because retained expertise cannot manually keep pace with constant change, knowledge must be externalized into curated artifacts, yet the validated query library must itself be actively maintained, a burden that the continuous analytic integration mechanism mitigates but does not remove.

## Implications and Future Research

The crisis of institutional amnesia in health care requires a structural shift. As long as analytical maturity is tied to individual tenure, organizations will remain fragile. By implementing HITL-KG, health systems can decouple intelligence from turnover, building a library of validated knowledge that ensures that maturity advances even as the workforce evolves.

Future research should empirically validate and refine the HITL-KG framework and the proposed 3-pillar assessment rubric. Priority questions include how pillar scores correlate with observed continuity of analytics performance during leadership and staff turnover; whether HITL-KG–mediated NL2SQL workflows reduce error rates, recovery time, and rework compared to baseline tooling; and which governance patterns most effectively balance safety, transparency, and equity when human validators operate at scale. Prospective multisite implementation studies, controlled user experiments, and qualitative implementation research will be needed to test these claims across diverse organizational, regulatory, and data environments.

## Supplementary material

10.2196/96541 Multimedia Appendix 1 Validated query triple examples illustrating the human-in-the-loop knowledge governance knowledge capture artifact. Each triple comprises a natural language intent, executable SQL, and rationale metadata for 3 clinical analytics scenarios: diabetes care monitoring, heart failure readmission cohort identification, and quality metric aggregation.

10.2196/96541 Multimedia Appendix 2 Three-pillar organizational assessment rubric: 9 indicators across analytics maturity, workforce agility, and technical enablement, each with low, medium, and high strength scoring anchors and supporting evidence from the literature.

## Abbreviations

AMAM: Analytics Maturity Assessment Model
EMR: electronic medical record
HIMSS: Healthcare Information and Management Systems Society
HITL-KG: human-in-the-loop knowledge governance
LLM: large language model
NL2SQL: natural language to SQL
