# Supplementary material for: Health Care Analytics Challenges: A 3-Pillar Framework Connecting Analytics Maturity, Workforce Agility, and Technical Enablement
Source: Interact J Med Res. 2026 Aug 31;15:e96541. doi: 10.2196/96541 (PMC13528883; doi:10.2196/96541)
Supplement: Supplementary file 1 [file ijmr-v15-e96541-s001.docx]

Multimedia Appendix

Samuel Thomas Harrold, Yuimedi, Inc.

August 2026

# Multimedia Appendix 1

## Validated Query Triple Examples for Health Care Analytics

This appendix illustrates the validated query triple artifact proposed by the HITL-KG framework (see the main paper’s section “The Solution: Externalization via Sociotechnical Artifacts”). Each triple comprises 3 components: (1) *natural language intent*, the clinical business question; (2) *executable SQL*, the technical implementation; and (3) *rationale metadata*, the contextual “why” behind the logic. Together, these components capture not just *what* query was run but *why* it was constructed that way, preserving institutional knowledge that would otherwise be lost during staff turnover.

## The Externalization Process

Each validated query triple is produced through a 5-step externalization cycle that captures tacit knowledge as a byproduct of routine analytics. These steps correspond to steps 1-5 of the 6-step validated query cycle shown in the main paper’s Figure 2; the sixth step, persistence, is the cumulative outcome of repeated cycles:

1. *Query generation*: A user asks a question in natural language; the AI proposes candidate SQL based on schema knowledge.
2. *Semantic translation*: The AI translates the SQL back into a natural-language explanation of what it will do.
3. *Expert validation*: The domain expert confirms or corrects this interpretation. This is the critical moment of externalization, where the human-in-the-loop step transforms validation into iterative knowledge capture.
4. *Artifact storage*: The validated triple is hashed and stored in organizational memory.
5. *Retrieval*: Future queries semantically match against this repository first, retrieving trusted human knowledge before attempting probabilistic generation.

The worked examples below show the artifacts this process produces on a synthetic database.

### Triple 1: Diabetes Care Monitoring

*Natural language intent:* “Show me all diabetic patients over 65 who had an HbA1c test in the last 6 months”

*Executable SQL:*

SELECT p.patient_id, p.age, l.test_date, l.result_value
FROM patients p
JOIN lab_results l ON p.patient_id = l.patient_id
JOIN diagnoses d ON p.patient_id = d.patient_id
WHERE d.icd10_code LIKE 'E11%' -- Type 2 Diabetes
 AND p.age > 65
 AND l.test_name = 'HbA1c'
 AND l.test_date >= DATE_SUB(CURRENT_DATE, INTERVAL 6 MONTH)

*Rationale metadata:* Aligned with NCQA HEDIS measures for Comprehensive Diabetes Care (CDC), monitoring HbA1c testing compliance in older adult populations. Uses ICD-10 E11% for type 2 diabetes specifically (not E10% for type 1) per the synthetic organization’s coding convention. Six-month window matches the HEDIS reporting period.

### Triple 2: Heart Failure Readmission Cohort

*Natural language intent:* “Identify patients discharged with heart failure in 2024 who had a readmission within 30 days, excluding those discharged to hospice. For each, calculate their max creatinine levels during the index stay.”

*Executable SQL:*

WITH IndexAdmissions AS (
 SELECT
 a.admission_id,
 a.patient_id,
 a.discharge_date,
 a.discharge_disposition
 FROM admissions a
 JOIN diagnoses d ON a.admission_id = d.admission_id
 WHERE d.icd10_code LIKE 'I50%'
 AND a.discharge_date BETWEEN '2024-01-01' AND '2024-12-31'
 AND a.discharge_disposition != 'HOSPICE'
),
Readmissions AS (
 SELECT
 i.patient_id,
 i.admission_id AS index_admission_id,
 r.admission_date AS readmission_date,
 DATEDIFF(r.admission_date, i.discharge_date) AS days_to_readmit
 FROM IndexAdmissions i
 JOIN admissions r ON i.patient_id = r.patient_id
 WHERE r.admission_date > i.discharge_date
 AND r.admission_date <= DATE_ADD(i.discharge_date, INTERVAL 30 DAY)
)
SELECT
 r.patient_id,
 r.index_admission_id,
 r.days_to_readmit,
 MAX(CASE
 WHEN l.unit = 'mg/dL' THEN l.val_num
 WHEN l.unit = 'mmol/L' THEN l.val_num / 88.4
 ELSE NULL
 END) as max_creatinine_mgdl
FROM Readmissions r
JOIN lab_events l ON r.index_admission_id = l.admission_id
WHERE l.itemid IN (50912, 50913)
GROUP BY r.patient_id, r.index_admission_id, r.days_to_readmit;

*Rationale metadata:* Supports CMS Hospital Readmissions Reduction Program (HRRP) tracking. Hospice exclusion per CMS 2025 rules (discharge disposition != ‘HOSPICE’). Creatinine unit normalization (mmol/L to mg/dL via /88.4) required because this synthetic lab system stores results in mixed units depending on the originating facility. Lab item IDs 50912 and 50913 are MIMIC-III creatinine codes; local implementations must map to their own lab dictionary.

### Triple 3: Quality Metric Aggregation

*Natural language intent:* “How many patients were readmitted within 30 days of discharge for heart failure?”

*Executable SQL:*

SELECT COUNT(DISTINCT r.patient_id) as readmission_count
FROM (
 SELECT a1.patient_id, a1.discharge_date, a2.admission_date
 FROM admissions a1
 JOIN admissions a2 ON a1.patient_id = a2.patient_id
 JOIN diagnoses d ON a2.admission_id = d.admission_id
 WHERE d.icd10_code LIKE 'I50%'
 AND a2.admission_date BETWEEN a1.discharge_date
 AND DATE_ADD(a1.discharge_date, INTERVAL 30 DAY)
 AND a1.admission_id != a2.admission_id
) r

*Rationale metadata:* Aligned with CMS HRRP requirements for organizational quality reporting. COUNT(DISTINCT patient_id) ensures each patient is counted once even if they have multiple readmissions. This is the aggregate metric version of Triple 2; the detailed cohort query should be run first to validate individual cases before reporting the aggregate number.

## How Triples Preserve Institutional Knowledge

In traditional analytics workflows, only the SQL (component 2) would be saved, if anything. The natural language intent (component 1) would exist only in an email or chat message. The rationale metadata (component 3), the most critical knowledge for institutional continuity, would exist only in the departing analyst’s memory.

When a new analyst inherits these triples, they receive not just executable code but the clinical reasoning, regulatory context, and institutional conventions that informed the query’s construction. This is the mechanism by which HITL-KG converts ephemeral analytical work into durable organizational memory.

## References

1. National Committee for Quality Assurance (NCQA). HEDIS measures and technical resources. 2025. Available from: https://www.ncqa.org/hedis/
2. Centers for Medicare and Medicaid Services (CMS). Hospital Readmissions Reduction Program (HRRP). 2025. Available from: https://www.cms.gov/medicare/payment/prospective-payment-systems/acute-inpatient-pps/hospital-readmissions-reduction-program-hrrp
