# Supplementary material for: Health Care Analytics Challenges: A 3-Pillar Framework Connecting Analytics Maturity, Workforce Agility, and Technical Enablement
Source: Interact J Med Res. 2026 Aug 31;15:e96541. doi: 10.2196/96541 (PMC13528883; doi:10.2196/96541)
Supplement: Supplementary file 2 [file ijmr-v15-e96541-s002.docx]

Multimedia Appendix

Samuel Thomas Harrold, Yuimedi, Inc.

August 2026

# Multimedia Appendix 2

## Three-Pillar Organizational Assessment Rubric

This appendix presents the full 3-pillar assessment rubric proposed in the main paper (section “The 3-Pillar Rubric”). Each indicator is scored as low, medium, or high strength using evidence-based anchors from the literature reviewed in the main paper’s section “The Evidence Base: 3 Pillars”. Organizations scoring predominantly “low strength” across multiple pillars face the self-reinforcing degradation cycle that the framework identifies as the central threat.

### Pillar 1: Analytics Maturity

| Indicator | Low strength | Medium strength | High strength | Evidence |
| --- | --- | --- | --- | --- |
| HIMSS AMAM stage | Stages 0-2: Fragmented data, limited reporting | Stages 3-4: Integrated warehouse, standardized definitions | Stages 5-7: Predictive analytics, AI integration | [1,2] |
| Self-service analytics | None; all analytics require IT intervention | Partial; BI tools available but underutilized | Widespread; clinical staff access data directly | [3,4] |
| AI and NL interface | No NL2SQL or conversational analytics | Pilot programs or evaluation underway | Natural language query capability deployed | [5,6] |

### Pillar 2: Workforce Agility

| Indicator | Low strength | Medium strength | High strength | Evidence |
| --- | --- | --- | --- | --- |
| First-year analytics staff turnover | >30% (high instability) | 15-30% | <15% (high stability) | [7,8] |
| Average leadership tenure | < 3 years | 3-5 years | > 5 years | [9] |
| Knowledge concentration | Critical expertise held by 3 or fewer individuals | Partial documentation; some cross-training | Distributed expertise; documented processes | [10,11] |

### Pillar 3: Technical Enablement

| Indicator | Low strength | Medium strength | High strength | Evidence |
| --- | --- | --- | --- | --- |
| Data access | SQL/technical expertise required for all queries | IT queue for complex queries; basic self-service | Natural language or visual query interfaces | [4,6] |
| Interoperability | Fragmented systems; manual reconciliation | Partial integration; some automated feeds | Unified data platform; real-time integration | [12,13] |
| Schema coupling | Hard-coded reports break on schema change | Partial semantic layer; some automated feeds | Semantic layer adapts; CI/CD detects drift | [14,15] |

## References

1.	HIMSS Analytics. *Analytics maturity assessment model (AMAM) global report. Healthcare Information and Management Systems Society*. HIMSS Analytics; 2024. <https://www.himss.org/maturity-models/amam/>

2.	Healthcare IT News. At HIMSS24 APAC, the Adoption Model for Analytics Maturity gets facelift. Published online 2024. <https://www.healthcareitnews.com/news/asia/himss24-apac-adoption-model-analytics-maturity-gets-facelift>

3.	Health Catalyst. The healthcare analytics adoption model: A roadmap to analytic maturity. Published online 2020. <https://www.healthcatalyst.com/learn/insights/healthcare-analytics-adoption-model-roadmap-analytic-maturity>

4.	Shahbaz M, Gao C, Zhai L, Shahzad F, Hu Y. Investigating the adoption of big data analytics in healthcare: The moderating role of resistance to change. *Journal of Big Data*. 2019;6(1). doi:[10.1186/s40537-019-0170-y](https://doi.org/10.1186/s40537-019-0170-y)

5.	Ziletti A, D’Ambrosi L. Retrieval augmented text-to-SQL generation for epidemiological question answering using electronic health records. *NAACL 2024 Clinical NLP Workshop*. Published online 2024. <https://arxiv.org/abs/2403.09226>

6.	Yuan C, Ryan PB, Ta C, et al. Criteria2Query: a natural language interface to clinical databases for cohort definition. *Journal of the American Medical Informatics Association*. 2019;26(4):294-305. doi:[10.1093/jamia/ocy178](https://doi.org/10.1093/jamia/ocy178)

7.	NSI Nursing Solutions. *2026 NSI National Health Care Retention & RN Staffing Report*. NSI Nursing Solutions; 2026. <https://www.nsinursingsolutions.com/documents/library/nsi_national_health_care_retention_report.pdf>

8.	Hackney A. *Onboarding New Hires in the Clinical Informatics and Practice Support (CIPS) Department With the Tiered Skills Acquisition Model (TSAM): A Program Evaluation*. PhD thesis. ProQuest Dissertations; 2024. <https://search.proquest.com/openview/9a3a9ef301c01ff0129d07f685b8643f/1>

9.	WittKieffer. *CIO Insights: The State of Healthcare IT Leadership*. WittKieffer; 2024. <https://api.wittkieffer.com/wp-content/uploads/2012/10/cio-insights-the-state-of-healthcare-it-leadership-wittkieffer-october-2024.pdf>

10.	Massingham PR. Measuring the impact of knowledge loss: A longitudinal study. *Journal of Knowledge Management*. 2018;22(4):721-758. doi:[10.1108/JKM-08-2016-0338](https://doi.org/10.1108/JKM-08-2016-0338)

11.	Foss NJ. The emerging knowledge governance approach: Challenges and characteristics. *Organization*. 2007;14(1):29-52. doi:[10.1177/1350508407071859](https://doi.org/10.1177/1350508407071859)

12.	Gal MS, Rubinfeld DL. Data Standardization. *NYU Law Review*. 2019;94(4):737-770. <https://www.nyulawreview.org/issues/volume-94-number-4/data-standardization/>

13.	Zhang Y, Callaghan-Koru JA, Koru G. The challenges and opportunities of continuous data quality improvement for healthcare administration data. *JAMIA Open*. 2024;7(3):ooae058. doi:[10.1093/jamiaopen/ooae058](https://doi.org/10.1093/jamiaopen/ooae058)

14.	Mannapur SB. Understanding data drift and concept drift in machine learning systems. *International Journal of Scientific Research in Computer Science, Engineering and Information Technology*. 2025;11(1):318-330. doi:[10.32628/CSEIT25111239](https://doi.org/10.32628/CSEIT25111239)

15.	Battula SKY. Adaptive Data Quality Management for Multi-Cloud Healthcare Warehouses: FHIR-Aware Semantics and Unsupervised Thresholding. *International Journal of Artificial Intelligence, Data Science, and Machine Learning*. 2025;6(4):218-226. doi:[10.63282/3050-9262.IJAIDSML-V6I4P130](https://doi.org/10.63282/3050-9262.IJAIDSML-V6I4P130)
